# Palliative treatment of generalized metastatic follicular carcinoma of thyroid after operation: A case report and literature review

**DOI:** 10.1097/MD.0000000000038237

**Published:** 2024-05-17

**Authors:** Han Zhao, Wenyan Kang, Jian Guan, Ying Wang

**Affiliations:** a Department of Medical Oncology, National Cancer Center/National Clinical Research Center for Cancer/Cancer Hospital&Shenzhen Hospital, Chinese Academy of Medical Sciences and Peking Union Medical College, Shenzhen, China; b Department of Radiology, National Cancer Center/National Clinical Research Center for Cancer/Cancer Hospital&Shenzhen Hospital, Chinese Academy of Medical Sciences and Peking Union Medical College, Shenzhen, China; c Department of Pathology, National Cancer Center/National Clinical Research Center for Cancer/Cancer Hospital & Shenzhen Hospital, Chinese Academy of Medical Sciences and Peking Union Medical College, Shenzhen, China.

**Keywords:** follicular carcinoma, Lenvatinib, targeted therapy, thyroid

## Abstract

**Rationale:**

Follicular carcinoma of thyroid is a rare pathological type of thyroid carcinoma, accounting for 4.5% of the total. At present, the main treatment methods include surgery, iodine therapy, thyroid hormone inhibitors, etc. Targeted drug therapy is very important for distant metastasis and iodine-refractory differentiated thyroid cancer.

**Patient concerns:**

This clinical case is a 51-year-old male patient with follicular carcinoma of thyroid.

**Diagnoses:**

After 7 years of total thyroidectomy, multiple distant metastasis occurred to bilateral lungs, bones, multiple lymph nodes, etc.

**Intervention:**

After multidisciplinary consultation in the department of oncology, thoracic surgery, nuclear medicine and other departments, he received targeted drug therapy of Lenvatinib.

**Outcomes:**

After 3 months, his condition was partially relieved, and his quality of life was significantly improved. After 11 months of treatment, the evaluated efficacy was still in remission.

**Lesson:**

Late metastatic thyroid cancer is faced with dilemma of radioiodine refractory after traditional treatment. This will provide further evidence for therapeutic intervention in similar patients in the future.

## 1. Introduction

Thyroid follicular carcinoma is defined as a malignant tumor originating from thyroid follicular epithelial cells and lacking the nuclear characteristics of papillary thyroid carcinoma (PTC). Thyroid follicular carcinoma often presents with the following gene mutations: RAS (50%), PPARG fusion (30%), PIK3CA (10%), PTEN (10%), and TERT promoter mutations (20%). Thyroid follicular carcinoma with only capsule invasion but no vascular invasion have a good prognosis, while tumors with vascular invasion can lead to hematogenous metastasis. It accounts for approximately 4.5% of thyroid cancer, with a 5-year survival rate of nearly 88% and a 10-year survival rate of 78%.^[1]^ Thyroid follicular carcinoma with TERT gene mutation has a poor prognosis. Although the overall malignancy of thyroid follicular carcinoma is relatively low, it still poses a threat to the patient life, health, and quality of life. Due to its low mortality rate and long survival period, standardized diagnosis, treatment, and follow-up are more necessary. The present study describes a case of thyroid follicular carcinoma and analyzes its related clinical and pathological features as well as drug treatment methods when it occurs multiple systemic metastases.

## 2. Case report

**Figure 1.**
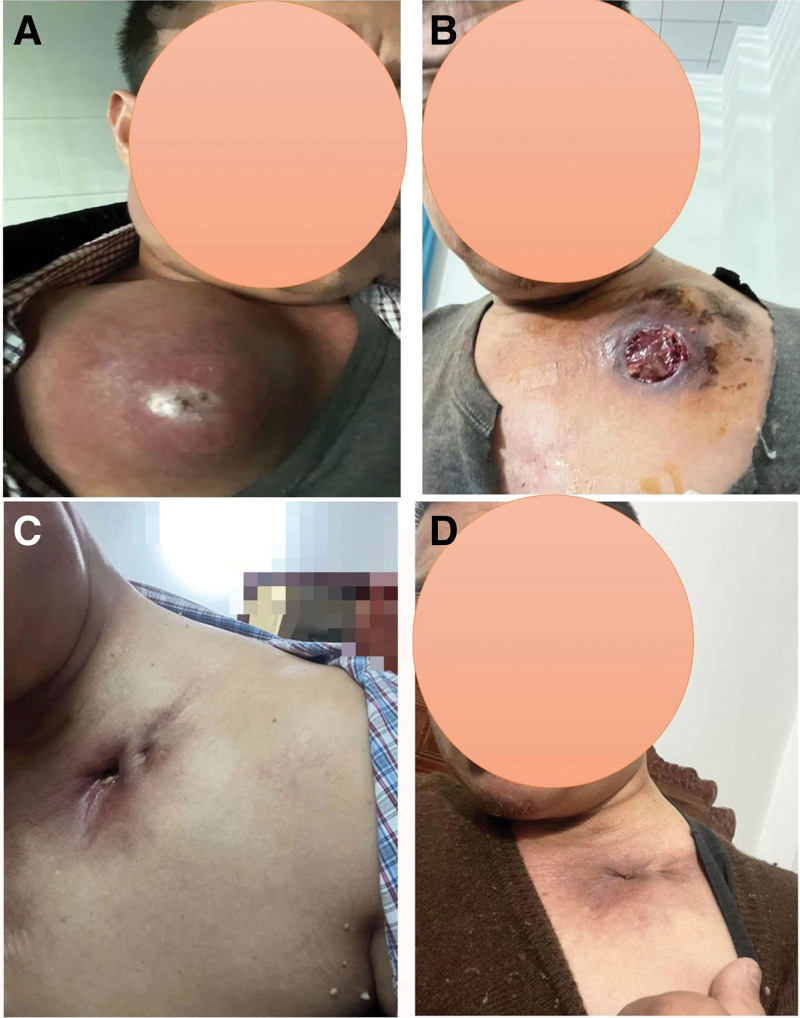
Appearance change of left anterior chest tumor: (A) before treatment (2022.01), the left anterior chest tumor was huge, and the patient head movement was limited. (B) Three months after treatment (2022.03), the left anterior chest tumor was significantly reduced, and the wound presented an ulcer about 6 to 7 cm in size. (C) Nine months after treatment (2022.09) and (D) nearly 1 yr after treatment (2022.12), the ulcer wound was basically healed.

**Figure 2.**
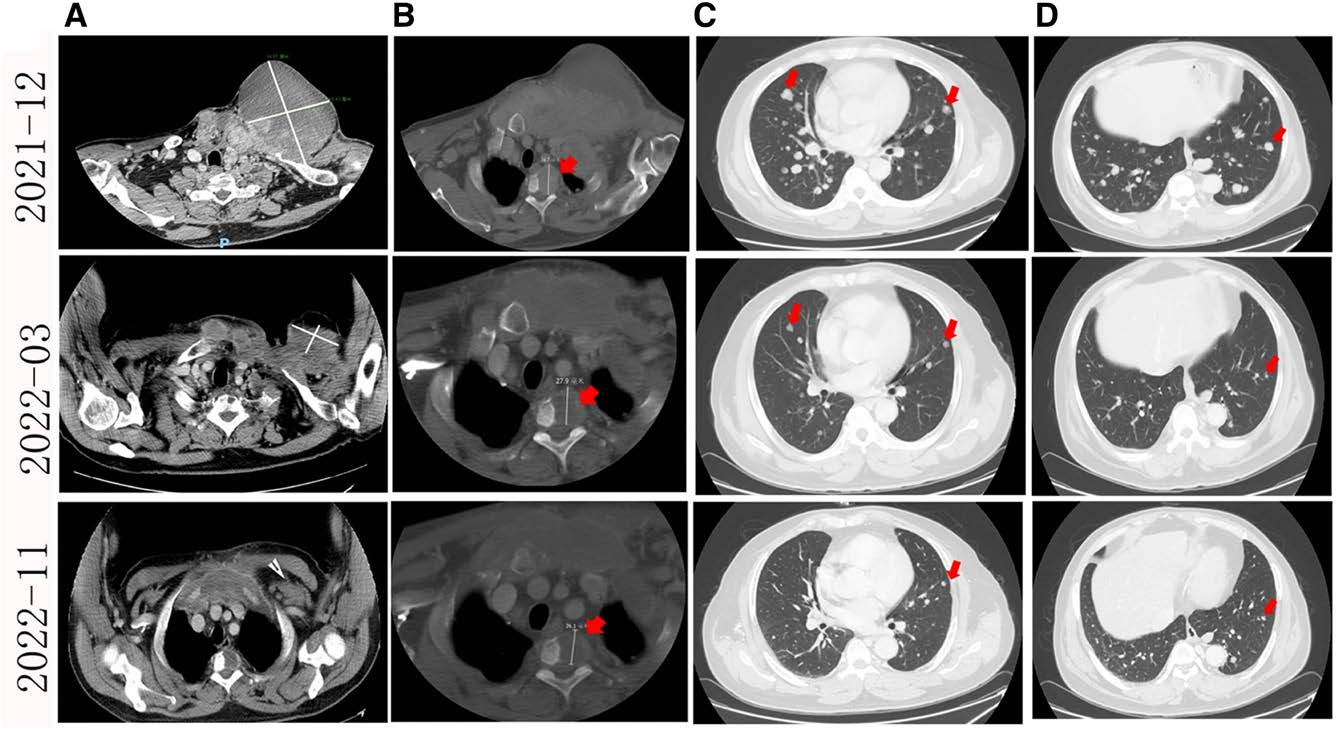
Evaluation results of 2021 to 2012, 2022 to 2003, and 2022 to 2011 CT (plain scan + enhanced scan) (typical lesions are shown as arrows). 2021 to 2012, (A) Left anterior chest tumor, about 14 * 10 cm in size; (B) Spinal bone metastasis; (C–D) Multiple metastatic tumors in both lungs, the larger one is about 2.5 * 1.8 cm. 2022 to 2003, (A) Left anterior chest tumor, about 12.5 * 6.3 cm in size; (B) Spinal bone metastasis; (C–D) Bilateral lung metastasis, the larger one is about 1.8 * 1.4 cm. 2022 to 2011, (A) Left anterior chest tumor, about 11.1 * 4.2 cm in size; (B) Spinal bone metastasis. (C–D) Bilateral lung metastasis, the larger one is about 1.7 * 1.2 cm.

**Figure 3.**
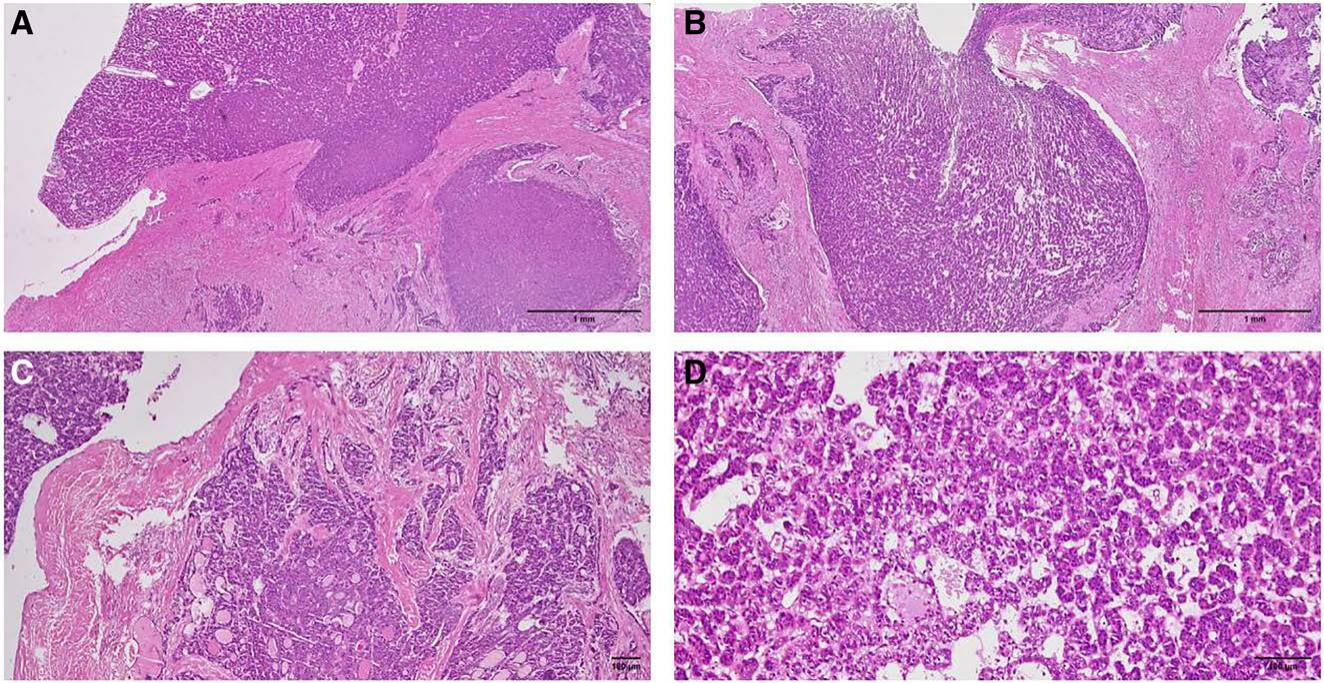
The pathomorphological characteristics of the surgical tissues of the primary thyroid tumor removed in 2014: (A) Tumor tissue involves the capsule and forms isolated tumor nests outside the capsule (magnification, 2×; hematoxylin and eosin staining; scale bar, 1 mm); (B) Tumor tissue invading the capsule and extracellular tissue (magnification, 4×; hematoxylin and eosin staining; scale bar, 1 mm); (C) Most of the tumor tissue presents follicular structure, with some lumens underdeveloped and some lumens containing concentrated glia (magnification, 4×; hematoxylin and eosin staining; scale bar, 100 µm); (D) The tumor cells are enlarged, nuclear Chromatin is deeply stained, with moderate heteromorphism, but the karyotype is relatively round, without papillary carcinoma karyotype, nuclear distortion and severe heteromorphism (magnification, 10×;d hematoxylin and eosin staining; scale bar, 100 µm).

The patient, a 51-year-old male, underwent total thyroidectomy in the external hospital in September 2014 due to “follicular carcinoma of thyroid.” Postoperative staging is T3bN0M0. After the operation, he received iodine-131 treatment. In 2018, the ultrasound showed the left supraclavicular mass, which was gradually increased after oral Chinese medicine treatment. In December 2021, he visited our hospital and be found an irregular huge mass in the left forechest (Fig. 1A). On December 29, 2021, CT reexamination showed an irregular mass in the thyroid bed and the anterior superior mediastinum, with the largest section of 13.4 * 3.9 cm, invading adjacent structures; Multiple lymph node metastasis occurred in bilateral neck, mediastinum, armpit, inner breast area and pectoral wall muscle space, with the size of about 2.9 * 2.7 cm; Multiple metastasis in both lungs and pleura; Multiple bone metastases (Fig. 2); The patient refused to undergo a full body PET-CT examination due to economic reasons. Based on the patient medical history and imaging data, the clinical diagnosis of the patient is follicular thyroid cancer with multiple systemic metastases. The consultation of previous surgical pathology showed follicular carcinoma of thyroid (Fig. 3). The slides of the thyroid tumor tissue were stained with hematoxylin and eosin at room temperature for 5 minutes. This case of follicular carcinoma of thyroid is not an early minimally invasive lesion, but a widely infiltrating one. The tumor tissue is extensively infiltrating and growing within fibrous connective tissue, so the small invasion of the capsule and vascular tumor thrombi presented in the early lesions are not obvious. The tumor tissue of this lesion shows differentiation of thyroid follicles, with a relatively round karyotype. There is no clear nuclear distortion or severe nuclear atypia, and there is no clear necrosis or island/solid structure. Therefore, the diagnosis is follicular carcinoma of thyroid, with extensive infiltration. Gene detection showed that ATM, NRAS, PMS1, SMARCA4, TERT mutations, TMB 8.22Must/MB, MSS. The DNA was extracted using the QIAamp DNA FFPE Tissue Kit(QIAGEN). Fragmented DNA was then end-repaired, A-tailed, and ligated to indexed adapters using the KAPA Hyper Prep Kit (Kapa Biosystems). Adapter-ligated libraries were purified using AMPure XP beads (Beckman Coulter) and quantified using a Qubit fluorometer (Thermo Fisher Scientific). The raw data shared on online repository (URL:https://www.ncbi.nlm.nih.gov/bioproject/PRJNA998481).

After multi-disciplinary-treatment consultation of nuclear medicine department and thoracic surgery department, it is not recommended to take iodine-131 treatment and local treatment such as surgery because of large tumor load and high bleeding risk. Since January 13, 2022, the targeted oral treatment of mesylate Lenvatinib has been started, with a dose of 20 mg qd for 6 weeks. Due to poor symptomatic treatment and control of hypertension, it has been adjusted to 16 mg qd for 12 weeks, and adjusted to 12 mg qd so far due to intolerance of hypertension. From March 2022 to now, physical examination showed that the left anterior chest tumor was significantly smaller than before (Fig. 1B–D). CT reexamination showed that the thyroid bed and the anterior superior mediastinum were irregular tumors, and the scope was smaller than before. Multiple enlarged lymph nodes were smaller than before. Multiple metastatic tumors in both lungs were smaller than before (Fig. 2); The evaluation result is partially relieved. The last follow-up time of the patient was April 2024 (Table 1), and the assessment result was still in remission (Fig. 2).

**Table 1:** Summary of timeline of the patient past medical history.

Treatment time	Therapy	Response evaluation	DFS or PFS
September 2014-October 2014	Total thyroidectomy and postoperative Iodine-131 treatment	NA	45 mo
June 2018-December 2021	Oral Chinese medicine treatment	PD	——
December 2021-April 2024	Oral mesylate Lenvatinib	PR	28 mo

PR = partial response.

## 3. Discussion

Thyroid cancer is the most common malignant tumor of endocrine system.^[2,3]^ In 2020, the number of new thyroid cancer cases in the world is about 580,000 and the incidence rate ranks 11th among all cancers.^[4]^ Thyroid carcinoma can be divided into differentiated carcinoma, medullary carcinoma and undifferentiated carcinoma according to its pathological type. Papillary carcinoma and follicular carcinoma are the main types of differentiated carcinoma with the highest incidence rate. PTC refers to malignant epithelial tumors originating from thyroid follicular epithelial cells with characteristic PTC nuclear characteristics. Thyroid follicular carcinoma is defined as a malignant tumor originating from thyroid follicular epithelial cells and lacking PTC nuclear characteristics. The pathological diagnosis of follicular carcinoma is difficult; It does not display cellular or nuclear atypia. Invasion of the tumor capsule or capsular blood vessels is the necessary condition of follicular carcinoma. At present, the main methods for differentiated thyroid cancer include surgery, iodine therapy, thyroid hormone inhibitors, etc. After iodine treatment, 5% to 23% of patients have distant metastasis, and 1/3 of patients with distant metastasis will gradually lose their differentiation and iodine uptake ability under natural conditions or during treatment, and eventually develop into radioiodine refractory differentiated thyroid cancer (RAIR-DTC).^[5]^ Targeted drug therapy plays a very important role in distant metastasis and RAIR-DTC, mainly including tyrosine kinase inhibitors, BRAF inhibitors and MEK inhibitors. At present, there are also studies proving that immunotherapy can benefit the survival of patients with advanced thyroid cancer.^[6,7]^ The prognosis of thyroid follicular carcinoma is similar to that of papillary carcinoma. If the tumor is confined to the thyroid, with a diameter of <1 cm, or has micrometastasis, both have good prognosis. If there is distant metastasis or high invasion, the prognosis is poor.

This patient had a huge tumor load at the recurrence site with ulceration and bleeding when he first visited our hospital; There was metastasis of lung, pleura, bone and other distant organs; The stage was late and the prognosis was poor. For personal reasons, since the onset of the disease, only oral Chinese medicine has been used for treatment in the past 2 years, and no systematic anti-tumor treatment has been received. The tumor has developed rapidly. The patient was generally tolerable to the oral targeted treatment of Lenvatinib. The main adverse reactions were grade III hypertension and grade I Hand-foot syndrome. After the dosage of Lenvatinib was gradually reduced from 20 mg to 12 mg, the adverse reaction could be alleviated. In the later follow-up period, the tumor on the body surface was significantly reduced and the quality of life was significantly improved. A case report in 2022 described a 77-year-old man with diagnosis of metastatic differentiated thyroid cancer and evidence of presence of mutation of BRAF K601E on liquid biopsy was treated with sorafenib, showing a good response to the treatment.^[8]^

A number of retrospective studies have confirmed the efficacy of Lenvatinib in the real world. Nervo et al^[9]^ retrospectively analyzed the effectiveness of Lenvatinib in 12 patients with RAIR-DTC. When the starting daily dose of Lenvatinib was 24 mg, after a median follow-up of 13.3 months, the 1-year progression-free survival rate was 54.6%, and the overall survival rate was 75.0%; Among the 12 patients, 5 patients achieved partial response (PR), and 2 patients were stable disease.

In addition, there are also some case reports that immunotherapy can benefit advanced thyroid cancer. One patient with MSI-H follicular thyroid cancer reached continuous PR after receiving treatment with Pembrolizumab.^[10]^ Another case of thyroid cancer with BRAF gene mutation benefited from the treatment of Nivolumab.^[11]^ The main reason why the case reported did not receive combined immunotherapy is that its gene detection is MSS type. At present, there are few clinical studies on immunotherapy in thyroid cancer, and there is still insufficient evidence.

At present, targeted therapy of thyroid cancer has been confirmed by many clinical studies. With the update of data from several clinical studies including COSMIC-311 (Cabozantinib), ALTER01032 (Anlotinib), LIBRETTO-001 (Selpercatinib), DECISION study (Sorafenib), REALITY study (Apatinib) in Phase III, the treatment of RAIR-DTC patients has gradually become chronic. The SELECT study on Lenvatinib show that it can improve the quality of life of patients while effectively treating RAIR-DTC, and give consideration to the efficacy and tolerance of patients in the real world.

There are also some studies have been confirmed on immunotherapy. KEYNOTE-158, a phase II clinical study, explored the effectiveness of Pembrolizumab in the treatment of MSI-H/dMMR advanced solid tumors.^[6]^ 233 cases of MSI-H/dMMR non-colon cancer patients were included in the study (including 5 cases of thyroid cancer). The efficacy analysis showed that the objective response rate was 34.3%, of which 23 cases (9.9%) reached complete remission (CR), and 57 cases (24.5%) reached PR. Another phase Ib study, KEYNOTE-028, included 22 patients with advanced differentiated thyroid cancer and explored the efficacy of Pembrolizumab.^[7]^ Only patients with PD L1 TPS ≥ 1% were included in this study. The median follow-up time was 31 months, and the median progression-free survival time was 7 months (95% CI: 2–14 months); The median total survival time was not reached, of which 2 cases (9%) reached PR and 12 cases (55%) were stable disease.

Differentiated thyroid cancer is faced with dilemma of iodine refractory after traditional treatment. The survival rate of iodine-refractory differentiated thyroid cancer is low, and the 10-year survival rate is only 10%. New therapies for iodine-refractory thyroid follicular carcinoma are needed. Since the end of the 20th century, clinical research on the molecular mechanism of thyroid cancer has opened the era of multi-target molecular therapy for thyroid cancer. At present, some studies have confirmed that differentiated thyroid cancer benefits from immunotherapy, but the evidence is limited, which needs to be confirmed by more large multicenter clinical studies to provide more options for the treatment of thyroid cancer patients. These include further exploration and improvement of redifferentiation therapy as well as development of targeted therapies, immunotherapies and combination therapies.

## Author contributions

**Validation:** Jian Guan.

**Visualization:** Wenyan Kang.

**Writing – original draft:** Han Zhao.

**Writing – review & editing:** Ying Wang.
